# Mechanistic Insights for Microbiome Application in Plant Disease Resistance

**DOI:** 10.1111/mpp.70233

**Published:** 2026-03-06

**Authors:** Jiakang Yin

**Affiliations:** ^1^ College of Life Sciences and Institute for Conservation and Utilization of Agro‐Bioresources in Dabie Mountains Xinyang Normal University Xinyang China

**Keywords:** biocontrol mechanisms, disease resistance, microbiome application, microbiome modulation, plant microbiome

## Abstract

Plant diseases caused by biotic and abiotic stresses pose a great threat to both plant health and yield. Plant microbiomes play a crucial role in improving disease resistance, representing a sustainable approach to enhance crop performance. Plant host factors, including genetic variation, metabolites and microRNA, shape the assembly and function of the plant microbiome, thereby augmenting disease resistance. This interplay presents opportunities for plant‐mediated manipulation of microbiome to promote plant health. Multiple mechanisms are involved in the microbiome‐mediated plant disease resistance, such as direct and indirect pathogen antagonism, niche pre‐emption, alteration of microbiota and activation of plant defences. Nevertheless, the application of plant microbiome in the field remains limited due to the intrinsic complexity of plant–microbiome and environment–microbiome interactions. This review synthesises current knowledge on the roles of plant microbiomes in plant disease resistance. I further summarise the mechanisms underlying plant‐guided microbiome modulation and probiotic‐mediated disease suppression. I also raise work and challenges that should be addressed, with the ultimate goal of informing more efficient microbiome application in sustainable agriculture.

## Graphical abstract

This review summarises the mechanisms underlying plant‐guided microbiome modulation and probiotic‐mediated disease suppression. Additionally, it highlights the challenges to be addressed for a better microbiome application of disease resistance in sustainable agriculture and proposes possible strategies. Figure was created with BioRender.

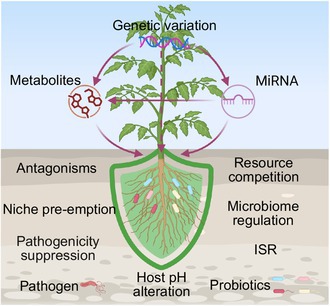

**Abbreviations**
2,4‐DTBP: 2,4‐di‐*tert*‐butylphenol
3‐OH PAME: 3‐hydroxypalmitic acid methyl ester
4‐HCA: 4‐hydroxycinnamic acid
B: 148 bacteria
BX: benzoxazinoid
CSR: corn stalk rot
DAPG: 2,4‐diacetylphloroglucinol
DIMBOA: 2,4‐dihydroxy‐7‐methoxy‐2*H*‐1,4‐benzoxazin‐3(4*H*)‐one
EPS: exopolysaccharide
ET: ethylene
F: 34 fungi
Foc: *Fusarium* *oxysporum* f. sp. *conglutinans*
GWAS: genome‐wide association study
ISR: induced systemic resistance
JA: jasmonic acid
MF: microbe‐free
M genes: host genes involved in modulating microbiota
miRNA: microRNA
NUE: nitrogen‐use efficiency
O: eight oomycetes
*rin*
: ripening‐inhibitor
SA: salicylic acid
SynComs: synthetic microbial communities
T3SS: type III secretion system
T6SS: type VI secretion system
WT: wild‐type

## 1 Background

The term microbiome encompasses both the microbiota (the community of microorganisms) and their ‘theatre of activity’, which includes structural elements, metabolites/signal molecules and the surrounding environmental conditions (Berg et al. 2020). The microbiome interacts intimately with environments, serving an indispensable function within the holistic One Health paradigm, an approach that integrates the health of humans, animals, plants and the environment (Law et al. 2024). Plant microbiomes refer to the entire microbiomes associated with plants, encompassing those in the phyllosphere (above‐ground components), rhizosphere (the soil zone influenced by root exudates) and root endosphere (inside the roots). They are considered to be the plant's second genome and can provide a range of beneficial functions, such as plant growth promotion, nutrition acquisition, stress tolerance, flavour improvement and disease resistance (Copeland et al. 2025). Meanwhile, they play negative roles in plant disease resistance (Chen et al. 2020; Li et al. 2022; Durán et al. 2018; Pan et al. 2025).

The assembly and function of plant microbiomes are influenced by various aspects, such as drought (Xu et al. 2018, 2021), pathogen invasion (Carrión et al. 2019; Berendsen et al. 2018; Wei et al. 2018), plant genetic variation (Zhang et al. 2019; Chen et al. 2020; Yin, Zhang, Zhu, et al. 2022; Su et al. 2024), plant metabolites (Su et al. 2024; Wang et al. 2025; Yang, Fu, et al. 2023) and plant microRNA (miRNA) (Liu et al. 2025). Among these, plant genetic variation, metabolites and miRNA have been shown to confer disease resistance by shaping microbiome, offering promising plant‐mediated avenues for microbiome‐based plant disease resistance. Beneficial microbes assist plants in defending against diseases through a variety of mechanisms, which include antagonism against pathogens directly or indirectly (Ping et al. 2024; Mendes et al. 2011; Fan et al. 2024; Yin, Zhang, Guo, et al. 2022; Liu et al. 2023; Sun et al. 2025), niche pre‐emption (Xia et al. 2024; Oszust et al. 2020), pathogenicity suppression (Yin, Zhang, Zhu, et al. 2022; Wang et al. 2023), resource competition (Gu et al. 2020; Wei et al. 2015), microbiome regulation (Yang, Zheng, et al. 2023; Tao et al. 2020; Zhang et al. 2025) and induced systemic resistance (ISR) (Berendsen et al. 2018; Ping et al. 2024; Lee et al. 2021). These mechanisms provide a foundation for designing rational and multifunctional synthetic microbial communities (SynComs) to enhance their application in sustainable agriculture.

In this review, I emphasise the important contributions of plant microbiomes in plant disease resistance. I then synthesise the mechanisms underlying host‐mediated microbiome assembly and function, as well as those by which beneficial microbes confer disease resistance. I also highlight in‐depth work and challenges that needs to be addressed for more efficient microbiome application.

## 2 Contributions of Plant Microbiomes in Plant Disease Resistance

### 2.1 Positive Contributions

Accumulating evidence demonstrates the positive effect of plant microbiomes in resisting plant diseases. Mendes et al. (2011) employed PhyloChip to dissect the rhizosphere microbiota of sugar beet plants grown in soils with different levels of suppressiveness against *Rhizoctonia solani*. They identified *Pseudomonadaceae*, *Burkholderiaceae*, *Xanthomonadales*, *Lactobacillaceae* and *Actinobacteria* as the key taxa associated with the suppression of disease caused by *R. solani* . By coupling culture‐dependent and random transposon mutagenesis approaches, they demonstrated that one isolate from *Pseudomonadaceae* protects sugar beet plants from *R. solani* infection through the production of a putative chlorinated lipopeptide encoded by nonribosomal peptide synthetase genes (Mendes et al. 2011). Similarly, in a more recent study, Xia et al. (2024) also highlights the positive role of plant microbiomes in resisting corn stalk rot (CSR). They investigated the microbiota associated with CSR‐susceptible and CSR‐resistant cultivars through multi‐omics approaches along with experimental validation. Their results demonstrated that *Bacillus* species enriched in the CSR‐resistant cultivar are the core microbiota conferring the suppression of CSR (Xia et al. 2024).

Beyond individual microbes, complex microbial communities have also been shown to protect plants from disease. Durán et al. (2018) assembled seven SynComs comprising 148 bacteria (B), 34 fungi (F), eight oomycetes (O) and their cross‐kingdom combinations (BO, BF, FO and BFO) and inoculated them on sterile *Arabidopsis thaliana* Col‐0. Their results showed that the B and BFO SynComs notably enhance the growth and survival rate of *A. thaliana* compared to microbe‐free (MF) plants, with the BFO consortium conferring a greater benefit, suggesting that multikingdom interactions yield more profound plant‐beneficial effects. Besides, they found that the B SynCom rescues the detrimental impacts on plant growth and survival rate exerted by the filamentous eukaryotic microbes (F, O and FO) (Durán et al. 2018). Moreover, Berendsen et al. identified that three bacterial species from *Xanthomonas*, *Microbacterium* and *Stenotrophomonas* are recruited by *A. thaliana* upon *Hyaloperonospora arabidopsidis* infection. These bacteria were found to defend against downy mildew caused by *H. arabidopsidis* by triggering an ISR synergistically (Berendsen et al. 2018).

### 2.2 Negative Contributions

Plant microbiomes can also exert negative effects on plant disease resistance. From the study of Durán et al. (2018) mentioned previously, the F, O and FO SynComs were demonstrated to be detrimental to the growth and survival rate of *A. thaliana* when compared to the MF control. Furthermore, an improperly assembled phyllosphere microbiota has also been shown to have an adverse impact on plant health. Chen et al. found that the *Arabidopsis* quadruple mutant (*mfec*), simultaneously defective in pattern‐triggered immunity and the MIN7 vesicle‐trafficking pathway, displays leaf damage when grown in non‐sterile soils, while the wild‐type (WT) Col‐0 does not. However, both genotypes grow well under sterile conditions. The endophytic leaf microbiota of the *mfec* mutant shows dysbiosis when compared with the WT Col‐0. The dysbiosis features highly enriched Proteobacteria and markedly depleted Firmicutes. By infiltrating a 48‐member bacterial community derived from Col‐0 (SynCom^Col‐0^) and a 52‐member bacterial community derived from *mefc* (SynCom^mfec^) into Col‐0 leaves, they found that SynCom^mfec^ rather than SynCom^Col‐0^ results in severe leaf necrosis and chlorosis (Chen et al. 2020). In a separate study, Li et al. (2022) screened 160 non‐redundant bacterial isolates derived from tomato rhizosphere and demonstrated that 26.9% of them inhibit *Ralstonia solanacearum* in vitro, while 50.6% of them promote *R. solanacearum* growth. More recently, Pan et al. demonstrated that the rhizosphere of rice varieties that are highly susceptible to rice false smut recruits *Sphingomonadaceae* microbes. These microbes, in turn, assist the fungal pathogen in breaking chlamydospore dormancy and colonising root tissues (Pan et al. 2025).

Therefore, a comprehensive approach to microbiome application in plant disease resistance should consider both beneficial and detrimental microbial components. A synergistic strategy that concurrently enriches beneficial taxa and suppresses pathogenic ones is likely to be more effective than targeting either group in isolation. Additionally, restoring dysbiotic microbiota to a homeostatic state represents a promising alternative strategy.

## 3 Host Control of Plant Microbiome Assembly and Function for Disease Resistance (Figure 1a)

**FIGURE 1:**
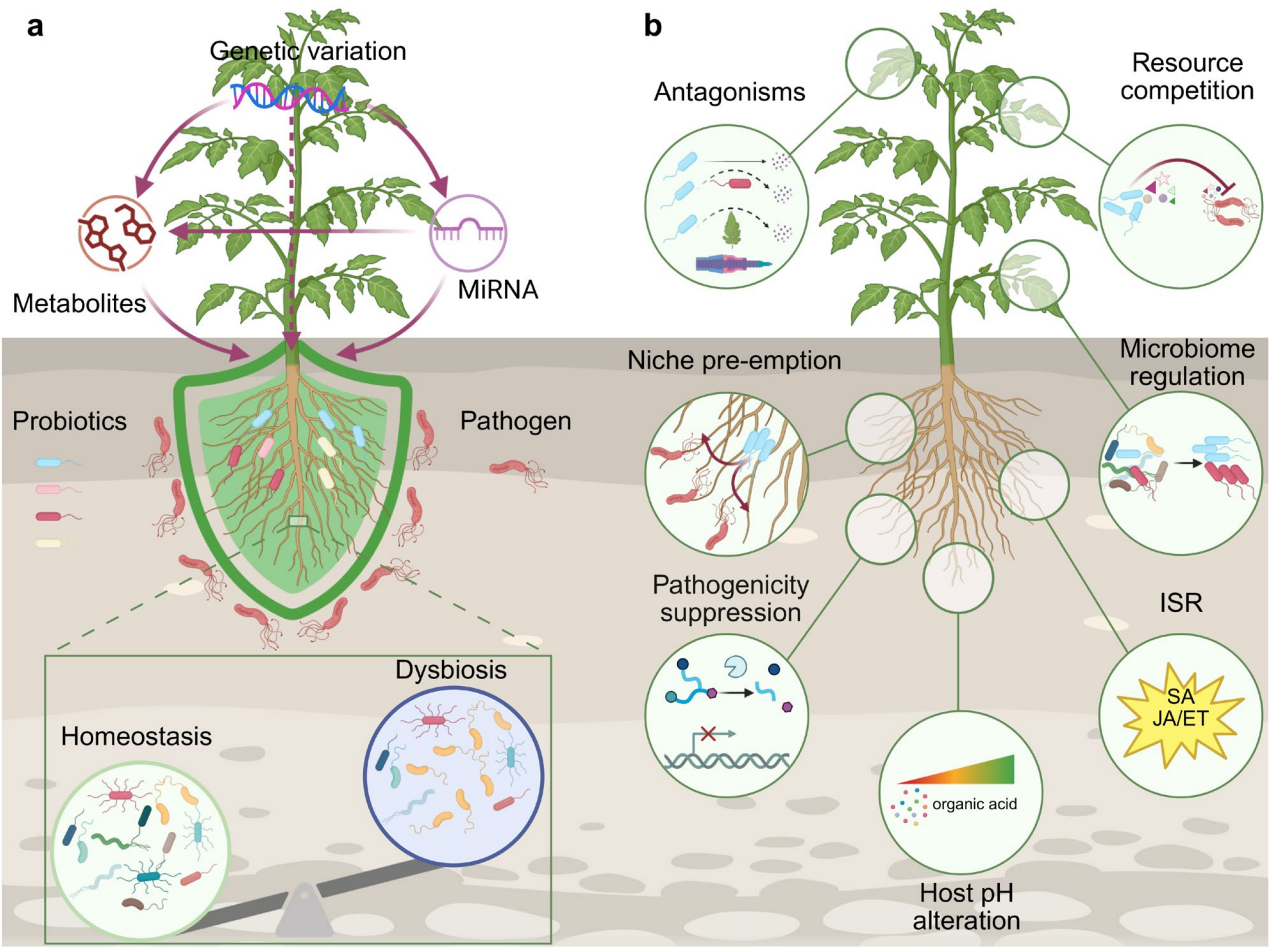
Microbiome‐mediated plant disease resistance. (a) Host regulation of microbiome for disease resistance. In the upper part, the solid lines represent established interactions, while dashed line indicates hypothesised relationship. (b) Mechanisms of biocontrol. Created with BioRender.

Numerous factors, as discussed above, influence the assembly and function of the plant microbiome. Here I focus on host‐associated factors that offer promising strategies for manipulating the plant microbiome in sustainable agriculture: plant genetic variation, plant metabolites and plant miRNA.

### 3.1 Plant Genetic Variation

The mechanism by which host genetic variation modulates the plant microbiome to enhance disease resistance has been extensively characterised. As described previously, *A. thaliana* mutant *mfec* harbours dysbiotic endophytic phyllosphere microbiota featured by an enriched Proteobacteria and a depleted Firmicutes compared to the WT Col‐0. In addition, the SynCom^mfec^ derived from the *mfec* mutant but not the SynCom^Col‐0^ derived from Col‐0 induces leaf damage on Col‐0 (Chen et al. 2020), which indicates that restoring a dysbiotic microbiota to homeostasis is a promising way of enhancing plant disease resistance. A study coupling multi‐omics and experimental verification identified that the rhizosphere of tomato cultivars highly resistant to bacterial wilt possesses distinct microbiota and recruits disease‐resistant bacteria *Sphingomonas* sp. Cra20 and *Pseudomonas putida* KT2440 compared to the susceptible ones. The ability to recruit bacterial wilt‐resistant rhizobacteria is heritable, as evidenced by the finding that both the resistant progeny HG64 and its resistant offspring HF12 recruit these two beneficial bacteria (Yin, Zhang, Zhu, et al. 2022). However, the genes responsible for this microbiota modulation remain to be elucidated. Despite the fact that the identification of host genes involved in modulating microbiota (referred to as *M* genes) has been challenging due to their polygenic nature (Copeland et al. 2025), successful cases have been reported. For example, based on 110 rice accessions, Su et al. (2024) performed a genome‐wide association study (GWAS) to investigate associations between rice genetic variations and its phyllosphere microbiome. They reported that most species identified by GWAS belong to *Pseudomonadales* (30.24%), *Burkholderiales* (21.37%), *Xanthomonadales* (8.87%) and *Enterobacterales* (8.67%). They further identified that a majority of peroxidase genes, which are involved in lignin biosynthesis, are associated with *Burkholderiales*, *Pseudomonadales* and *Xanthomonadales*. Using a gene‐editing approach including gene knockout and overexpression, they demonstrated that the *OsPAL02* gene regulates the abundance of specific microbial taxa, enriching *Pseudomonadales* while depleting *Burkholderiales* and *Xanthomonadales*. This gene encodes a phenylalanine/tyrosine ammonia‐lyase that catalyses the biosynthesis of 4‐hydroxycinnamic acid (4‐HCA) and *trans*‐cinnamic acid, which are both precursors for the biosynthesis of lignin. Furthermore, 4‐HCA together with *Pseudomonadales* provide protective effect against disease (Su et al. 2024). This report provides insights into targeted modulation of microbiome for plant disease resistance.

GWAS is a powerful tool for identifying genes that modulate the microbiome and thereby enhance disease resistance. However, GWAS requires a large panel of diverse accessions and entails significant costs. An alternative strategy involves utilising genes known to function in plant disease resistance, as demonstrated by Zhang et al. (2019). Given that *indica* varieties of rice harbour superior nitrogen‐use efficiency (NUE) genes compared to *japonica*, they characterised the root microbiota of 68 *indica* and 27 *japonica* varieties grown in the field and found that *indica*‐enriched bacterial taxa contain more genera with nitrogen metabolism functions than *japonica*‐enriched taxa. Subsequently, they focused on *NRT1.1B*, a nitrate transporter and sensor that has been reported to contribute to the difference in NUE of *indica* and *japonica* varieties. They demonstrated a key role of *NRT1.1B* in the recruitment and abundance of a large proportion of *indica*‐enriched bacteria in the rice root microbiota. More importantly, they found that *indica*‐enriched SynCom promotes plant growth more effectively than *japonica*‐enriched SynCom when organic nitrogen is the sole nitrogen source (Zhang et al. 2019).

*M* genes represent promising targets for microbiome‐mediated resistance breeding. Nevertheless, the performance of *M* genes and their effects on the plant microbiome under complex field conditions remain to be fully elucidated, which is essential for assessing their broad application potential. There may be other adverse functions of *M* genes beyond regulating the microbiome, which should be carefully examined to ensure a more balanced application in sustainable agriculture.

### 3.2 Plant Metabolites

Plant metabolites play a key role in shaping the plant microbiome, thereby improving disease resistance. For instance, by employing WT tomato and its isogenic ripening‐inhibitor (*rin*) mutant (less resistant to bacterial wilt than the WT tomato), riboflavin and 3‐hydroxyflavone enriched in the WT play a key role in disease suppression through recruiting disease‐suppressing *Streptomyces* (Yang, Fu, et al. 2023). These *Streptomyces* exhibit antagonistic activity against the pathogen *R. solanacearum* in vitro and reduce the disease severity of *rin* tomato plants (Yang, Fu, et al. 2023). In addition, as previously mentioned, by GWAS‐assisted method, Su et al. (2024) identified an *M* gene *OsPAL02* responsible for enriching *Pseudomonadales* while depleting *Burkholderiales* and *Xanthomonadales* and for producing 4‐HCA, which is important in disease resistance. 4‐HCA supports the growth of most tested *Pseudomonadales* strains but inhibits the growth of all tested *Burkholderiales* and *Xanthomonadales* strains. The presence of 4‐HCA together with *Pseudomonadales* exerts considerable suppression of disease. This study demonstrated that 4‐HCA‐mediated microbiome regulation is pivotal in rice phyllosphere homeostasis, which is helpful for rice disease resistance (Su et al. 2024). Wang et al. (2025) screened 23 root exudates for their ability to suppress tomato bacterial wilt and identified succinic acid as the most effective. Succinic acid does not inhibit the pathogen or affect tomato plants directly; instead, it suppresses the disease by reshaping the rhizosphere microbiome. Specifically, the supplementation of succinic acid leads to the enrichment of *Sphingomonas* sp. WX113. While this strain exhibits a pronounced biocontrol effect by itself, its efficacy is further enhanced when combined with succinic acid. Furthermore, they demonstrated that succinic acid boosts both nutrient competition and antagonistic activity of WX113 against the pathogen (Wang et al. 2025).

Apart from these above plant metabolites, the well‐known benzoxazinoid (BX) 2,4‐dihydroxy‐7‐methoxy‐2*H*‐1,4‐benzoxazin‐3(4*H*)‐one (DIMBOA) has also been shown to recruit potentially beneficial microbe that contributes to disease resistance. Employing BX WT and DIMBOA‐deficient *bx1* mutant maize plants, Neal et al. (2012) demonstrated that DIMBOA stimulates rhizosphere colonisation by *P. putida* KT2440. This strain has been reported to be disease‐suppressive and to trigger ISR (Matilla et al. 2010; Yin, Zhang, Zhu, et al. 2022; Bernal et al. 2017). They revealed that KT2440 exhibits tolerance to DIMBOA through metabolism‐dependent degradation. Furthermore, they confirmed KT2440's chemotactic motility towards DIMBOA (Neal et al. 2012). Root‐secreted cucurbitacin B has been demonstrated to modulate the rhizosphere microbiome by selectively enriching *Enterobacter* and *Bacillus*, which in turn results in robust resistance against the soil‐borne wilt fungal pathogen *Fusarium oxysporum* (Zhong et al. 2022). Interestingly, in vitro assays showed that the cucurbitacin B inhibits the growth of *Bacillus* while it stimulates the *Enterobacter*. The *Enterobacter* has positive effect on the growth of the *Bacillus*. Thus, the cucurbitacin B fuels the *Enterobacter*, which improves the growth of *Bacillus* (Zhong et al. 2022).

These reports shed light on applying plant metabolites as prebiotics based on their microbiome‐modulating ability. Nevertheless, future work is needed to evaluate the efficacy of potential prebiotics under varying agricultural contexts, consisting of different plant genotypes, resident soil microbiomes and climates, with the aim of identifying broad‐spectrum prebiotics applicable across diverse agricultural systems.

### 3.3 Plant miRNA

Plant miRNAs are a set of non‐coding RNAs that function in plant developmental programmes and responses to biotic/abiotic cues (Millar 2020). Additionally, plant miRNAs have been shown to contribute to disease resistance by shaping the plant microbiome. For example, tomato plants treated with organic fertiliser exhibit greater suppression of bacterial wilt compared to those treated with chemical fertiliser, a phenomenon associated with an enrichment of sly‐miR159 (Liu et al. 2025). Further analysis combining the rhizosphere microbiome data revealed that sly‐miR159 is positively correlated with two well‐known *R. solanacearum* ‐antagonising bacteria *Streptomyces* and *Bacillus* (Sun et al. 2023; Yang, Fu, et al. 2023), whose growth was demonstrated to be promoted by sly‐miR159 in vitro (Liu et al. 2025). However, the underlying mechanisms by which sly‐miR159 regulates these key bacteria remain to be elucidated. Work on plant miRNA‐mediated microbiome regulation for disease suppression is still in its infancy. Advancing this field is a crucial step towards the practical application of miRNA‐based strategies.

Overall, a conceptual model is proposed to elucidate how plants regulate their microbiome to enhance disease resistance (Figure 1a). This model illustrates that genetic variation in plants may exert its influence through metabolites, miRNA, miRNA‐metabolites flow or independently. miRNA can function either via modulating metabolite pathways or through its own direct regulatory mechanisms.

## 4 Mechanisms Underlying Probiotics‐Conferred Disease Resistance (Figure 1b)

### 4.1 Antagonism

The antagonism of probiotics against pathogens, which involves various molecules and mechanisms, is a well‐established strategy for plant disease resistance. Lipopeptides represent a well‐known class of these molecules. As a representative lipopeptide, fengycin is produced by several *Bacillus subtilis* strains and exhibits antagonistic activity against various fungi, including the pathogens *R. solani* and *Phytophthora palmivora* (Vanittanakom et al. 1986; Jacques et al. 1999). Its mechanism of action involves a two‐state transition: a monomeric, surface‐bound but non‐perturbing state, and a buried, aggregated form that causes leakage and confers bioactivity (Deleu et al. 2008). As noted earlier, one disease‐suppressive bacterial strain of *Pseudomonadaceae* directly antagonises the pathogen *R. solani* using a putative chlorinated lipopeptide encoded by nonribosomal peptide synthetase genes (Mendes et al. 2011). *B. subtilis* R31, which significantly reduces the incidence of tomato bacterial wilt, directly inhibits the growth of *R. solanacearum* . The production of lipopeptides including the fengycin, iturin and surfactin families is involved in this inhibitory process (Sun et al. 2023).

Using genetic and metabolomic approaches, a study revealed that 2,4‐diacetylphloroglucinol (DAPG) produced by the root‐associated *Pseudomonas brassicacearum* R401 is also partially involved in the inhibitory activity against *R. solanacearum* (Getzke et al. 2023). Further genome‐based evaluation demonstrated an increased abundance of DAPG biosynthetic gene cluster in root‐derived *Pseudomonas* isolates (Getzke et al. 2023). Antagonistic molecules have been identified not only in bacteria but also in fungi. It has been demonstrated that the fungus *Aspergillus cvjetkovicii*, enriched in the phyllosphere of disease‐resistant rice, suppresses the pathogen *R. solani* by producing 2,4‐di‐*tert*‐butylphenol (2,4‐DTBP). This compound scavenges reactive oxygen species, which in turn switche off the bZIP‐activated transcription of *AMT1*—a gene that positively regulates the growth of *R. solani* (Fan et al. 2024).

Apart from molecules, nanomachines are also key for antagonising pathogens in a contact‐dependent way. For instance, the type IV secretion system (T6SS) of *Lysobacter enzymogenes* OH11 is crucial to kill the pathogen *Pectobacterium carotovorum* , thereby protecting carrot plants from infection (Shen et al. 2021). Bernal et al. (2017) also showed that *P. putida* KT2440 protects *Nicotiana benthamiana* from the infection of *Xanthomonas campestris* by antagonism in a T6SS‐dependent way.

The mechanisms by which beneficial microbes antagonise pathogens remain to be fully elucidated. For example, *Chitinophaga eiseniae* , which is enriched in the healthy tomato‐growing soil compared to the soil with bacterial wilt, exhibits a direct inhibitory effect against *R. solanacearum* via unknown mechanisms (Yin, Zhang, Guo, et al. 2022). Moreover, after infection by soil‐borne fungus *Fusarium oxysporum* f. sp. *conglutinans* (Foc), the resistant cabbage variety YG selectively enriches *P. brassicacearum* NA13, which displays a notable Foc‐inhibiting effect via unclear mechanisms (Ping et al. 2024). Furthermore, as mentioned above, Xia et al. (2024) found *Bacillus* to be the core microbiota conferring the suppression of CSR, and direct antagonism against the pathogen *F. graminearum* is one of the mechanisms, though the molecular basis remains unclear. Uncovering these unknown mechanisms will not only deepen our fundamental understanding of microbial antagonism but also guide us towards more effective probiotics. For example, this knowledge could enable the engineering of probiotics capable of hyperproducing key inhibitory compounds.

Beyond direct antagonism, beneficial microbes can also antagonise pathogens indirectly. A study by Liu et al. (2023) found that isolates from *Aspergillus* and *Lactobacillus*, which are enriched in false‐smut‐suppressive rice panicles compared to diseased ones, effectively suppress the disease. The suppression relies on host leucine rather than on the direct antagonistic activity of the isolates. After successful colonisation, these isolates elevate the level of leucine, which has been demonstrated to antagonise the pathogen *Ustilaginoidea virens* by inducing apoptosis‐like cell death through H_2_O_2_ overproduction (Liu et al. 2023). Furthermore, the keystone bacterium *Paenibacillus cellulositrophicus* boosts the antagonistic capacity of *Streptomyces* R02 against *R. solanacearum* , despite having no direct antagonistic effect itself. It achieves this by inducing *Streptomyces* R02 to produce erythromycin E, a key antimicrobial compound that directly suppresses *R. solanacearum* (Sun et al. 2025). The identification of the molecules responsible for indirect antagonism in these probiotics is crucial for improving their biocontrol effects.

### 4.2 Niche Pre‐Emption

In biocontrol, niche pre‐emption refers to the phenomenon where a biocontrol agent suppresses a pathogen by colonising and occupying the niche before the pathogen can, preventing its establishment or spread. For example, fungi in the genus *Trichoderma* occupy an ecological niche similar to that of pathogenic *Colletotrichum* spp., which enables them to competitively exclude the later pathogen (Oszust et al. 2020). Bacteria have also been reported to confer disease resistance by niche pre‐emption. As mentioned above, Xia et al. (2024) identified *Bacillus* as the core microbiota conferring the suppression of CSR. This suppression involves, in part, niche pre‐emption, though the molecular mechanisms underlying this competition require further elucidation. Elucidating these details will guide the development of more efficient probiotics with enhanced competitive ability.

### 4.3 Pathogenicity Suppression

The pathogenicity is determined by various factors. For example, exopolysaccharide (EPS) is a major virulence determinant in *R. solanacearum* (Denny 1991). Its production is regulated by the quorum‐sensing autoinducer 3‐hydroxypalmitic acid methyl ester (3‐OH PAME) of *R. solanacearum* (Flavier et al. 1997; Shinohara et al. 2007). By using 3‐OH PAME as the sole carbon source to culture microorganisms from *Casuarina equisetifolia* branches and forest soil, Wang et al. (2023) identified *Pseudomonas forestsoilum* and *P. tohonis* as highly efficient degraders of this signal molecule. These two bacteria exhibit high esterase activity, inhibit EPS production in *R. solanacearum* , and protect *C. equisetifolia* , peanut and tomato plants from infection by *R. solanacearum* (Wang et al. 2023). Genes responsible for the hydrolysis of 3‐OH PAME and reduction of EPS production in *R. solanacearum* have also been uncovered in soil metagenome (Lee et al. 2018). The type III secretion system (T3SS) is also essential for the pathogenicity of *R. solanacearum* (Vailleau and Genin 2023). *Leifsonia aquatica* , which is enriched in healthy tomato‐growing soil, decreases bacterial wilt disease incidence by significantly suppressing the expression of the T3SS genes *hrpB* and *hrcC* in the pathogen *R. solanacearum* (Yin, Zhang, Guo, et al. 2022). In addition, the heritable rhizobacteria *Sphingomonas* sp. Cra20 and *P. putida* KT2440 protect tomato from bacterial wilt by employing multiple strategies, including suppressing the expression of virulence genes (i.e., EPS and T3SS genes) in the pathogen *R. solanacearum* (Yin, Zhang, Zhu, et al. 2022). The molecular bases for this suppression of virulence that help targeted probiotics engineering are still yet to be discovered.

### 4.4 Resource Competition

Some probiotics confer disease resistance by resource competition. For example, using five non‐virulent but closely related *Ralstonia* spp. strains derived from tomato rhizosphere, Wei et al. (2015) assembled 31 bacterial communities covering all possible species richness and community compositions. They investigated the effect of these communities on the invasion resistance to the pathogen *R. solanacearum* . Their results showed that communities with low nestedness, high connectance and high nutritional utilisation overlapping with the pathogen protect tomato from the disease (Wei et al. 2015), indicating the importance of resource competition in disease resistance. Iron is a crucial element participating in many major biological processes, such as trichloroacetic acid cycle, oxygen transport, gene regulation and DNA biosynthesis (Andrews et al. 2003). However, its available form is scarce (Colombo et al. 2014). Siderophores are compounds for scavenging iron from the environment, which are essential in plant disease resistance (Hider and Kong 2010; Calvente et al. 2001; Gu et al. 2020). For instance, Gu et al. (2020) found that the rhizosphere bacteria producing growth‐inhibitory siderophores often suppress the pathogen *R. solanacearum* in vitro as well as in natural and greenhouse soils, and protect tomato plants from infection. Additionally, *Bacillus subtilis* CAS15 confers the reduction of Fusarium wilt incidence in a siderophore‐dependent manner (Yu et al. 2011).

### 4.5 Microbiome Regulation

*Bacillus*, as a well‐known probiotic, can also assist in resisting plant diseases through microbiome regulation. For example, *Bacillus cereus* AR156, which was isolated from forest soil, displays effective control of tomato bacterial wilt. It is unable to antagonise the pathogen *R. solanacearum* directly. Instead, it can promote the enrichment of beneficial microorganisms in the plant rhizosphere by regulating salicylic acid (SA) and jasmonic acid (JA)/ethylene (ET) signalling pathways in plants, thereby playing a role in controlling bacterial wilt disease (Yang, Zheng, et al. 2023). Additionally, bio‐organic fertiliser presents considerable control effect over Fusarium wilt disease of banana. *Bacillus amyloliquefaciens* W19 in the bio‐organic fertiliser has been demonstrated to be crucial for the control effect. The control effect is associated with the impacts on the resident soil microbial communities, specifically an increase of specific *Pseudomonas* spp. Subsequent pot experiments demonstrated that the *Pseudomonas* strain PSE82 helps W19 reduce the density of pathogen *Fusarium oxysporum* f. sp. *cubense* (Tao et al. 2020). However, molecules in these bacteria responsible for the recruitment of beneficial bacteria remain to be identified. Not only bacteria but also fungi can enhance disease suppression through modulating the plant microbiome. As demonstrated by a recent study, Zhang et al. (2025) revealed that when infected by *Botrytis cinerea*, plants transfer JA via common mycorrhizal networks that are formed by arbuscular mycorrhizal fungi to neighbouring plants. This chemical signal induces shifts in the root exudates of the receiver plants, thereby promoting the recruitment of specific, disease‐suppressive microbial taxa such as *Streptomyces* and *Actinoplanes*.

### 4.6 ISR

ISR is an enhanced state of plant resistance primed by beneficial microbes. It is typically characterised by its dependence on the JA/ET signalling pathways rather than the SA pathway, which is associated with systemic acquired resistance (Choudhary et al. 2007). However, multiple studies have shown that both SA and JA/ET signalling pathways are activated in ISR (Niu et al. 2011; Samaras et al. 2021; Yuan et al. 2019). One of the best‐characterised ISR‐inducing probiotics is *Pseudomonas fluorescens* WCS 417r, which can offer protection against the fungal pathogen *Alternaria brassicicola* and bacterial pathogen *X. campestris* pv. *armoraciae* (Ton et al. 2002). Another versatile *Pseudomonas* strain *P. putida* KT2440, which has been demonstrated to protect plants from diseases by T6SS‐dependent antagonism and virulence suppression (Bernal et al. 2017; Yin, Zhang, Zhu, et al. 2022), can also offer protection through ISR (Matilla et al. 2010). *Bacillus*, a well‐known probiotic genus, has also been reported to confer protection through ISR (Niu et al. 2011; Xie et al. 2019). In particular, *B. cereus* AR156 can not only confer bacterial wilt disease suppression in tomato through microbiome modulation, but also provides protection from *Pseudomonas syringae* pv. *tomato* in *Arabidopsis* (Niu et al. 2011). The fungus *Trichoderma longibrachiatum* H9 has also been shown to lead to ISR in cucumber, thereby inhibiting the foliar pathogen *B. cinerea* infection (Yuan et al. 2019).

Beyond individual microbes, microbial consortia also exhibit the ability to activate ISR, thereby enhancing disease resistance. Notably, the constituent members often function synergistically. For instance, the three bacterial species from *Xanthomonas*, *Microbacterium* and *Stenotrophomonas*, which are enriched upon the infection by downy mildew pathogen *H. arabidopsidis*, defend disease by activating ISR synergistically (Berendsen et al. 2018). Additionally, the SynCom comprising *Brevibacterium frigoritolerans* HRS1, *Bacillus niacini* HRS2, *Solibacillus silvestris* HRS3 and *Bacillus luciferensis* HRS4, which are specifically enriched in the healthy tomato rhizosphere soil without bacterial wilt disease, displays protection from disease by ISR (Lee et al. 2021). The protection and ISR offered by the SynCom are greater in comparison with each individual strain (Lee et al. 2021).

Lots of microbial elicitors of ISR inducers, which mainly include *Bacillus* and *Pseudomonas* strains, have been discovered, such as antibiotics (e.g., DAPG), lipopeptides, siderophores (e.g., pipercide and pyoverdine), volatiles (e.g., 2,3‐butanediol and acetoin) and rhamnolipids (Kour et al. 2024). *P. putida* extracellular haem‐peroxidase (PP2561) has also been identified as essential for ISR (Matilla et al. 2010).

### 4.7 pH Alteration of Host

A more recent study reported that probiotics‐conferred disease defence is related to host pH alteration (Xu et al. 2025). By leveraging wheat head microbial community profiling, Xu et al. found that *Pseudomonas* spp. are enriched on wheat heads upon *Fusarium graminearum* infection. Furthermore, using experimental validation and metatranscriptomics, they demonstrated that specific *Pseudomonas* strains inhibit *F. graminearum*‐induced pH upshift via organic acids, thereby conferring the control of wheat Fusarium head blight (Xu et al. 2025). These host‐acidifying *Pseudomonas* strains are enriched during *F. graminearum* invasion (Xu et al. 2025).

## 5 Conclusions and Future Perspectives

Substantial evidence, as synthesised in this review, underscores the plant microbiome as a critical determinant of plant health, offering a versatile and sustainable arsenal for disease management. I have explored the dual roles of microbiomes, where specific taxa and consortia can confer robust disease resistance through a multitude of mechanisms—including direct and indirect antagonism, niche pre‐emption, pathogen virulence suppression, resource competition, microbiome remodelling, ISR and host pH alteration. Conversely, the presence of deleterious microbes or dysbiotic communities can exacerbate disease, highlighting the importance of a balanced microbial equilibrium for plant health.

A pivotal insight is the profound influence of the host plant on its microbial inhabitants. Host genetic variation, metabolites and miRNA act as powerful levers, shaping the assembly and function of the microbiome to enhance disease resistance. This plant‐mediated regulation provides a promising strategy for precision microbiome manipulation, moving beyond the external application of probiotics towards cultivating a naturally suppressive plant‐associated environment.

**FIGURE 2:**
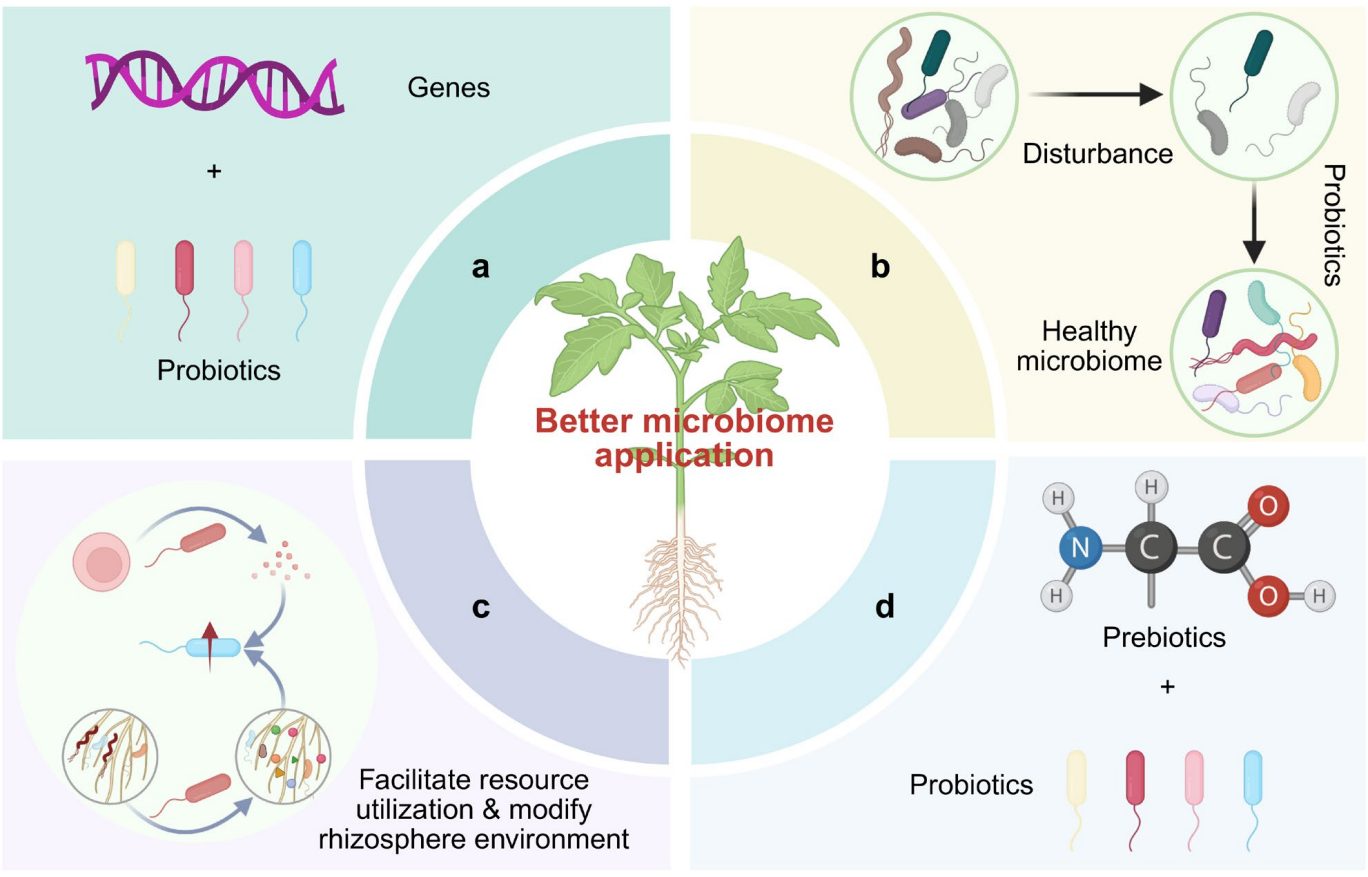
Promising strategies for efficient microbiome application in plant disease resistance. (a) Combination of key genes and probiotics for robust biocontrol effciency. (b) Disturbance of the resident microbial community, thereby enabling probiotic effects. (c) SynCom‐based competitiveness improvement of the introduced probiotics through facilitation of resource utilisation and rhizosphere environment modification. (d) Combination of probiotics and prebiotics, referred to as synbiont, for enhanced biocontrol efficacy. Created with BioRender.

Despite the compelling potential, the transition of microbiome‐based strategies from laboratory to field is constrained by several challenges:

(1) A primary challenge is the prevalent genotype‐dependency of biocontrol effects (Yin, Zhang, Zhu, et al. 2022; Vogel et al. 2021; Matilla et al. 2010; Su et al. 2024), which hinders the application of microbiome in practice. Determining the genes responsible for the efficient biocontrol effects is necessary for successful disease resistance. Furthermore, combination of the probiotics and these genes might provide robust protection among various genotypes or even species (Figure 2a).

(2) Moreover, given that plant metabolites can be modified in a microbiome‐dependent way and the microbiome is climate‐dependent (Thoenen et al. 2023; Sun et al. 2014; Liang et al. 2015), the performance of plant metabolites‐mediated microbiome regulation in disease resistance across planting regions remains largely unknown. Dissecting the exact interactions between plant metabolites and microbiome will help to predict and enhance the disease defence efficacy.

(3) Finally, biocontrol efficacy is often inconsistent in agricultural practices (Bardin et al. 2015). Successful colonisation of the introduced microbe is a fundamental prerequisite for exerting its biocontrol effects. This colonisation process is governed by resident microbiome, biotic interactions and host exudates. Hence, a primary possibility for this inconsistency is the failure of probiotics to overcome the resident microbiome, which is jointly shaped by plant genotype and environmental factors (e.g., via root morphology and exudates and creating different environmental niches) (Negre Rodríguez et al. 2025), thereby failing to establish and be effective. Therefore, perturbing the indigenous microbiome before applying probiotics may offer us an alternative (Figure 2b). As reported by Deng et al. (2021), after the drastic disturbance of initial state of microbiome by fumigation, both organic and bio‐organic fertiliser exhibited strong inhibition of wilt disease. Their study suggests that the fumigation ensures the development of an alternative community composition that develops into a healthy soil microbiome (Deng et al. 2021). Alternatively, colonisation may fail due to a lack of supportive biotic interactions within the resident community. More complex microbial co‐occurrence networks are often positively correlated with enhanced plant disease resistance (Ge et al. 2021; Yin, Zhang, Zhu, et al. 2022), and metabolic complementarity among microbes has been demonstrated to improve plant disease resistance (Yang, Yang, et al. 2025). Therefore, designing sophisticated, multikingdom SynComs represents a strategy to overcome this by providing built‐in beneficial interactions, thereby improving the ecological competence and colonisation success of introduced probiotics through facilitating resource utilisation and modifying the rhizosphere environment (Figure 2c). Another possibility is that the probiotics are not being supported by prebiotics, leading to colonisation failure. Hence, synbiotics, which is a combination of probiotics and prebiotics (Vyas and Ranganathan 2012), may offer elevated colonisation and biocontrol effects (Figure 2d). As indicated in a recent study, Yang et al. identified the probiotics *Pseudomonas* and prebiotics amino acids that are induced by the long‐term invasion of aboveground insect herbivore in cabbage rhizosphere. The amendment of amino acids enhanced the relative abundance of *Pseudomonas*. The cabbage plants exhibit the strongest resistance to insect herbivore when applied with the synbiotic, which combines the *Pseudomonas* and amino acids (Yang, Bezemer, et al. 2025).

## Author Contributions

**Jiakang Yin:** conceptualization, funding acquisition, visualization, writing – original draft, writing – review and editing.

## Funding

This work was supported by Xinyang Normal University Scientific Research Start‐up Fund. 2025 Key Pilot Research Projects in Natural Sciences, Xinyang Normal University, 5250046.

## Conflicts of Interest

The author declares no conflicts of interest.

## Data Availability

Data sharing not applicable to this article as no datasets were generated or analysed during the current study.
